# In Vitro Antibiofilm Effect of N-Acetyl-L-cysteine/Dry Propolis Extract Combination on Bacterial Pathogens Isolated from Upper Respiratory Tract Infections

**DOI:** 10.3390/ph16111604

**Published:** 2023-11-14

**Authors:** Dragana D. Božić, Ivana Ćirković, Jovica Milovanović, Biljana Bufan, Miljan Folić, Katarina Savić Vujović, Bojan Pavlović, Ana Jotić

**Affiliations:** 1 Department of Microbiology and Immunology, Faculty of Pharmacy, University of Belgrade, Vojvode Stepe 450, 11221 Belgrade, Serbia; biljana.bufan@pharmacy.bg.ac.rs; 2 Institute of Microbiology and Immunology, Dr Subotića 1, 11000 Belgrade, Serbia; cirkoviciv@yahoo.com; 3 Faculty of Medicine, University of Belgrade, Dr Subotića 8, 11000 Belgrade, Serbia; jmtmilov@gmail.com (J.M.); mfolic@yahoo.com (M.F.); katarinasavicvujovic@gmail.com (K.S.V.); pavlovicboj@gmail.com (B.P.); anajotic@yahoo.com (A.J.); 4 Clinic for Otorhinolaryngology and Maxillofacial Surgery, University Clinical Center of Serbia, Pasterova 2, 11000 Belgrade, Serbia; 5 Department of Pharmacology, Clinical Pharmacology and Toxicology, Dr Subotica 1, 11129 Belgrade, Serbia

**Keywords:** NAC/dry propolis extract fixed combination, antibiofilm effect, chronic rhinosinusitis, chronic otitis media, chronic adenoiditis, bacteria

## Abstract

Bacterial biofilms play an important role in the pathogenesis of chronic upper respiratory tract infections. In addition to conventional antimicrobial therapy, N-acetyl-L-cysteine (NAC) and propolis are dietary supplements that are often recommended as supportive therapy for upper respiratory tract infections. However, no data on the beneficial effect of their combination against bacterial biofilms can be found in the scientific literature. Therefore, the aim of our study was to investigate the in vitro effect of N-acetyl-L-cysteine (NAC) and dry propolis extract in fixed combinations (NAC/dry propolis extract fixed combination) on biofilm formation by bacterial species isolated from patients with chronic rhinosinusitis, chronic otitis media, and chronic adenoiditis. The prospective study included 48 adults with chronic rhinosinusitis, 29 adults with chronic otitis media, and 33 children with chronic adenoiditis. Bacteria were isolated from tissue samples obtained intraoperatively and identified using the MALDI-TOF Vitek MS System. The antimicrobial activity, synergism, and antibiofilm effect of NAC/dry propolis extract fixed combination were studied in vitro. A total of 116 different strains were isolated from the tissue samples, with staphylococci being the most frequently isolated in all patients (57.8%). MICs of the NAC/dry propolis extract fixed combination ranged from 1.25/0.125 to 20/2 mg NAC/mg propolis. A synergistic effect (FICI ≤ 0.5) was observed in 51.7% of strains. The majority of isolates from patients with chronic otitis media were moderate biofilm producers and in chronic adenoiditis they were weak biofilm producers, while the same number of isolates in patients with chronic rhinosinusitis were weak and moderate biofilm producers. Subinhibitory concentrations of the NAC/propolis combination ranging from 0.625–0.156 mg/mL to 10–2.5 mg/mL of NAC combined with 0.062–0.016 mg/mL to 1–0.25 mg/mL of propolis inhibited biofilm formation in all bacterial strains. Suprainhibitory concentrations ranging from 2.5–10 mg/mL to 40–160 mg/mL of NAC in combination with 0.25–1 mg/mL to 4–16 mg/mL of propolis completely eradicated the biofilm. In conclusion, the fixed combination of NAC and dry propolis extract has a synergistic effect on all stages of biofilm formation and eradication of the formed biofilm in bacteria isolated from upper respiratory tract infections.

## 1. Introduction

Biofilm represents a complex sessile community of microorganisms, irreversibly attached to the substrate and surrounded by a matrix of extracellular polymers. The biofilm structure allows bacteria to tolerate adverse environmental conditions, develop resistance to antimicrobial agents and the host immune system, and provides an optimal environment for the exchange of extracellular DNA [1,2,3]. Bacterial biofilms play an important role in the pathogenesis and persistence of many chronic and recurrent upper respiratory tract infections, including chronic rhinosinusitis, adenoiditis, and otitis media [4,5,6]. These conditions are the most common reason for the use of antimicrobials in children and adults worldwide and significantly affect the daily functioning and quality of life of the affected individuals [7].

Most available antimicrobial treatments are generally developed and evaluated against planktonic microorganisms. Bacteria in biofilms are significantly more resistant to the inhibitory and bactericidal effects of single and combined antibiotics than planktonic cells, therefore other treatment strategies should be explored [8]. There is ongoing interest in what compounds could be used to inhibit and eliminate bacterial biofilms. Besides conventional antimicrobial therapy, N-acetyl-L-cysteine (NAC) and propolis are supplements often recommended as adjuvant therapy in upper respiratory tract infections. NAC is a thiol antioxidant that acts as a precursor for glutathione and a mucolytic that hydrolyzes the disulfide bonds of mucus proteins [9]. NAC has previously been reported to inhibit biofilm formation by suppressing adhesion and matrix production or promoting the spread of already formed biofilms [10]. Propolis is considered a beneficial adjuvant therapy in respiratory infections with antibacterial, antioxidant, anti-inflammatory, and anti-proliferative properties, but its effect on biofilm formation has only recently been explored [11,12]. The chemical composition of propolis is complex and includes more than 300 identified chemical components. Its biological activity is mainly associated with flavonoids and phenolic acids [13,14]. Propolis has antimicrobial activity against planktonic forms as well as an inhibitory effect on the biofilm production of *Staphylococcus aureus* (both methicillin-susceptible (MS) and methicillin-resistant (MR), *Staphylococcus epidermidis*, and *Pseudomonas aeruginosa* [15,16,17]. Both NAC and propolis showed synergistic effects with certain antibiotics when tested as a single compound (propolis with beta-lactam antibiotics and NAC with cephalosporin) demonstrating the ability to enhance their effects even against resistant strains, which is a promising and effective strategy against bacterial biofilms [18,19]. However, the synergistic effect of a fixed combination of NAC and dry propolis extract against bacterial biofilms has not yet been studied.

Therefore, the aim of this study was to investigate the in vitro effect of NAC/dry propolis extract fixed combination (200 mg NAC/80 mg dry propolis extract) on the formation of biofilms by bacterial species isolated intraoperatively from tissue samples of patients with chronic upper respiratory tract infections (rhinosinusitis, adenoiditis, and otitis media).

## 2. Results

The study included 110 patients: 48 adults with chronic rhinosinusitis, 29 adults with chronic otitis media, and 33 children with chronic adenoiditis. The average age of the patients with chronic rhinosinusitis was 45.3 years (from 18 to 63 years), 42.7 years for chronic otitis media (from 18 to 66 years), and 5.6 years for children with chronic adenoiditis (from 3 to 16 years). There was no statistically significant difference between men and women, except in patients with chronic adenoiditis: 63.6% of men versus 36.4% of women (*p* < 0.05).

### 2.1. Bacterial Isolates

**Figure 1:**
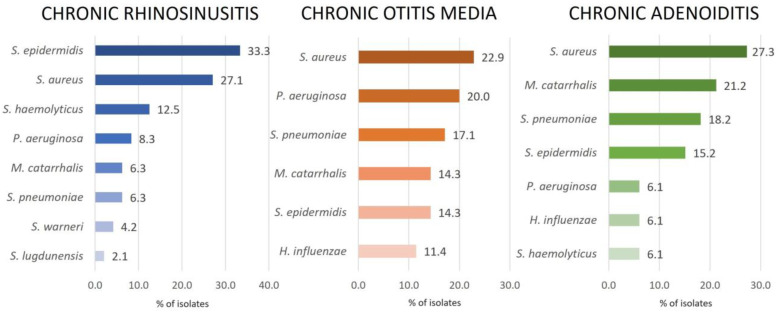
Distribution of bacterial isolates in various upper respiratory tract infections.

A total of 116 different strains were isolated from the tissue samples collected during the surgical procedure, which could be assigned to nine different bacterial species. The most abundant bacteria were staphylococci (57.8% of isolates), including coagulase-negative staphylococci (CNS): *Staphylococcus epidermidis*, *Staphylococcus haemolyticus*, *Staphylococcus lugdunensis*, and *Staphylococcus warneri* accounted for 31.9% and *Staphylococcus aureus* for 25.9% of all isolates, followed by *Moraxella catarrhalis* and *Streptococcus pneumoniae* with 12.9% of isolates each, *Pseudomonas aeruginosa* with 11.2%, and *Haemophilus influenzae* with 5.2% of total isolates. Frequency and species of isolates varied according to the specific pathology (Figure 1). Staphylococci were the most frequently isolated bacteria in patients with chronic rhinosinusitis (79.1%), chronic otitis media (37.2%), and chronic adenoiditis (48.6%).

### 2.2. Antimicrobial Activity of NAC/Dry Propolis Extract Fixed Combination

**Figure 2:**
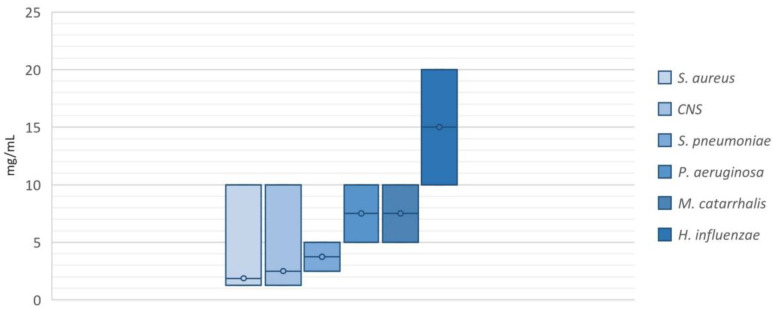
Minimum, maximum and average MIC values for each species. The average MIC values are shown as lines within the columns.

MICs of the NAC/dry propolis extract fixed combination ranged from 1.25/0.125 to 20/2 mg NAC/mg propolis. The best antimicrobial activity was observed against *S. aureus* (average MIC 1.87/0.187 mg NAC/mg propolis) and CNS (average MIC 2.50/0.25 mg NAC/mg propolis). Slightly higher MIC values were observed for *S. pneumoniae* (average MIC 3.75/0.375 mg NAC/mg propolis), *P. aeruginosa*, and *M. catarrhalis* (both with an average MIC of 7.50/0.75 mg NAC/mg propolis), and the highest MIC values were observed for the species *H. influenzae* (average MIC 15/1.5 mg NAC/mg propolis). The minimum, maximum, and average MIC values for each species are shown in Figure 2.

### 2.3. Synergistic Effect of NAC/Dry Propolis Extract Fixed Combination

**Figure 3:**
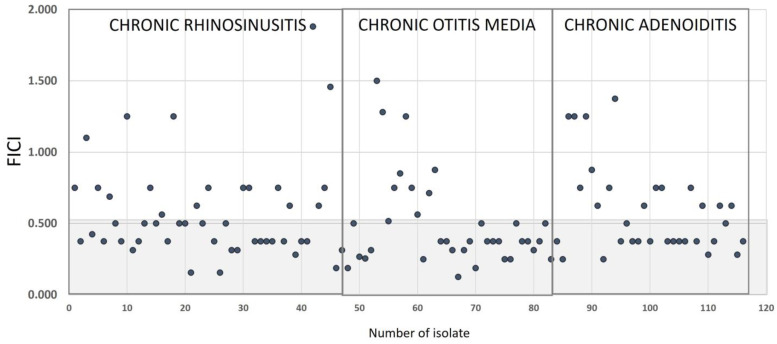
Distribution of FICI indices of NAC/dry propolis extract combination in strains isolated from the patients with chronic rhinosinusitis, chronic otitis media, and chronic adenoiditis. Numbers on the x-axis indicate the number of strains isolated, with isolates coded 1–48 (chronic rhinosinusitis); 49–83 (chronic otitis media); and 84–116 (chronic adenoiditis) (see Supplementary Files, Table S1, for information on strain identification and isolate code numbers). The number of strains obtained from patients with chronic otitis media (35) does not match the number of patients (29) because six of them had two isolates. The dots inside the gray area represent the strains in which the combination of NAC/dry propolis extract had a synergistic effect.

The MICs of the individual components of the NAC/dry propolis extract fixed combination were two to four times higher than their MICs in the fixed combination. The MIC for NAC decreased from 2.5–80 mg/mL alone to 1.25–20 mg/mL in combination with propolis, and the MIC of propolis decreased from 0.25–8 mg/mL to 0.125–2 mg/mL. FICI values ranged from 0.125 to 1.881. A synergistic effect (FICI ≤ 0.5) was observed in 51.7% of strains, mainly *S. aureus* (15.5%), *S. epidermidis* (12.1%), and *S. pneumoniae* (9.5%). Indifferent interactions (FICI > 0.5) were observed in 48.3% of strains, most frequently in *S. aureus* and *S. epidermidis* (both 10.3%) and *M. catarrhalis* and *P. aeruginosa* (both 9.5%). None of the interactions were in the antagonism range (FICI > 4). Applying other criteria defined by Hu et al. (additive effect for 0.5 < FICI ≤ 1) [20], 37.9% of the strains were found to have an additive effect and only 10.3% of the interactions were in the range of indifference. The distributions of FICI indices in isolates from different pathologies are shown in Figure 3.

### 2.4. Biofilm Production

**Figure 4:**
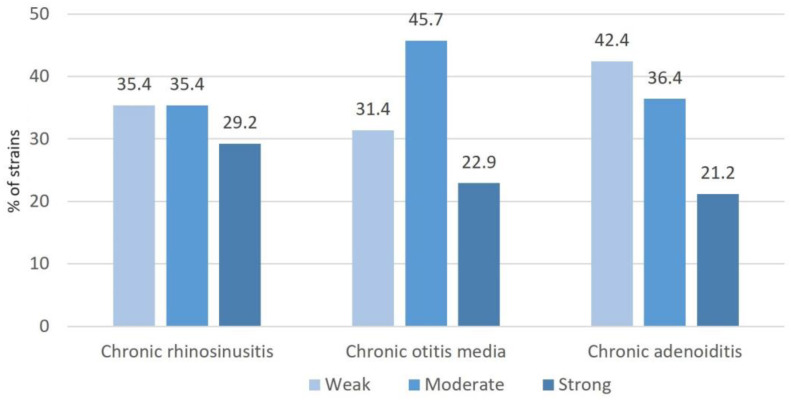
Biofilm production capacity of bacteria isolated from surgical tissues of patients with chronic rhinosinusitis, chronic otitis media and chronic adenoiditis.

The in vitro biofilm production capacity of strains isolated from different upper respiratory tract pathologies is shown in Figure 4.

The majority of isolates obtained from patients with chronic otitis media were moderate biofilm producers (category 2, *p* < 0.05); in patients with chronic adenoiditis they were weak biofilm producers (category 1, *p* < 0.05), while in patients with chronic rhinosinusitis, the same percentage of isolates were weak and moderate biofilm producers (categories 1 and 2).

### 2.5. Effect of NAC/Dry Propolis Extract Combinations on Biofilm Formation and Biofilm Eradication

#### 2.5.1. Chronic Rhinosinusitis

**Figure 5:**
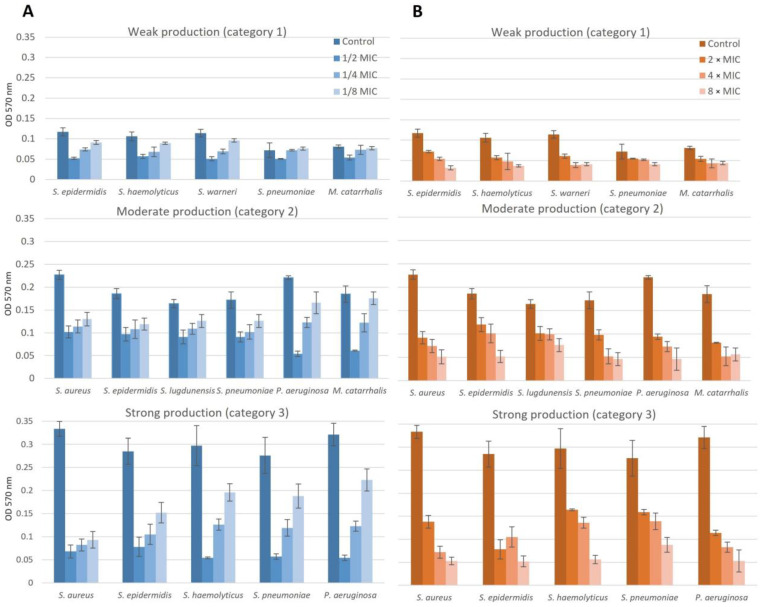
Dose dependency of NAC/dry propolis extract combinations on biofilm formation (**A**) and eradication of a previously formed biofilm (**B**) in isolates obtained from patients with chronic rhinosinusitis.

The effect of NAC/dry propolis extract on biofilm formation and biofilm eradication in isolates obtained from patients with chronic rhinosinusitis is shown in Figure 5.

Subinhibitory concentrations reduced biofilm formation at all applied concentrations (*p* < 0.05). The effect was dose-dependent; a concentration of 1/2 MIC completely prevented biofilm formation in 72.9% of isolates, and 1/4 MIC in 18.8% of isolates. A concentration of 1/8 MIC did not completely inhibit biofilm production, but reduced biofilm production from strong or moderate (categories 2 and 3) to weak (category 1) in 62.5% of isolates.

Suprainhibitory concentrations reduced the amount of previously formed biofilm at all concentrations applied (*p* < 0.05). The effect was dose-dependent, a concentration of 2 × MIC completely eradicated biofilm in 22.9% of isolates and reduced the category of biofilm production in 66.7% of isolates, and a concentration of 4 × MIC eradicated biofilm in 47.9% of isolates or reduced its category in the same percentage of isolates. The 8 × MIC concentration was the most effective, completely eradicating biofilm in 81.3% of isolates or reducing its category in all other isolates (*p* < 0.05).

#### 2.5.2. Chronic Otitis Media

**Figure 6:**
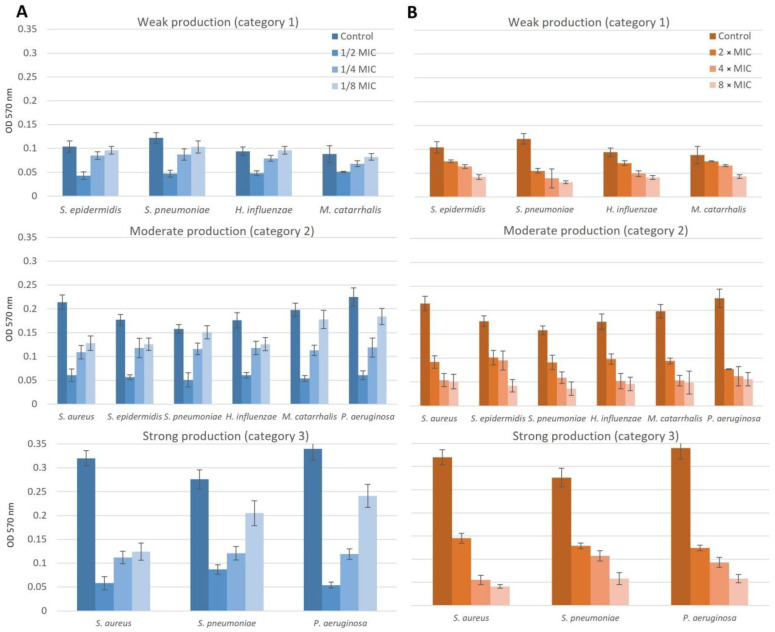
Dose dependency of NAC/dry propolis extract combinations on biofilm formation (**A**) and eradication of biofilm (**B**) in isolates obtained from patients with chronic otitis media.

The inhibitory effect on biofilm formation and eradication in isolates obtained from patients with chronic otitis media is shown in Figure 6.

Subinhibitory concentrations reduced biofilm formation at all applied concentrations (*p* < 0.05). The effect was dose-dependent, a concentration of 1/2 MIC completely prevented biofilm formation in 80.0% of isolates, and a concentration of 1/4 MIC prevented biofilm formation in 14.3% of isolates. A concentration of 1/8 MIC did not completely inhibit biofilm production, but most isolates (57.4%) significantly reduced biofilm production from strong or moderate (categories 2 and 3) to weak (category 1).

Suprainhibitory concentrations reduced preformed biofilm at all concentrations applied (*p* < 0.05). The effect was dose-dependent: a concentration of 2 × MIC completely eradicated biofilm in 17.1% of isolates and reduced the category of biofilm production in 74.3% of isolates, and a concentration of 4 × MIC eradicated biofilm in 48.6% of isolates and reduced the category in 34.3% of isolates. The most effective concentration was 8 × MIC, which resulted in complete biofilm eradication in 91.4% of isolates and category reduction in an additional 5.7% of isolates (*p* < 0.05).

#### 2.5.3. Chronic Adenoiditis

**Figure 7:**
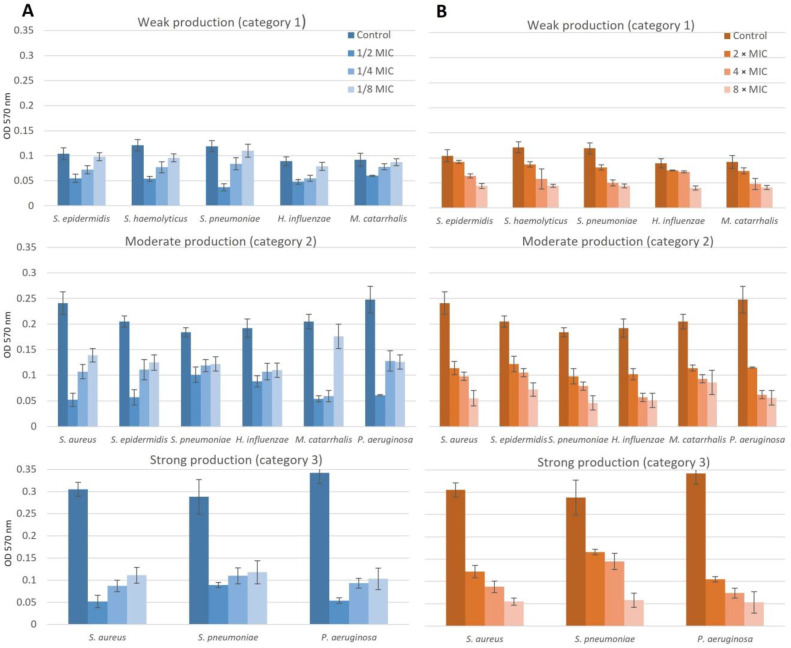
Dose dependency of NAC/dry propolis extract combinations on biofilm formation (**A**) and eradication of previously formed biofilm (**B**) in isolates obtained from patients with chronic adenoiditis.

The inhibitory/eradication effect of NAC/dry propolis extract combinations on biofilms in isolates obtained from patients with chronic adenoiditis is shown in Figure 7.

Subinhibitory concentrations reduced biofilm formation at all applied concentrations (*p* < 0.05). The effect was dose-dependent: a concentration of 1/2 MIC completely prevented biofilm formation in 81.8% of isolates, and a concentration of 1/4 MIC prevented biofilm formation in 30.3% of isolates. A concentration of 1/8 MIC did not completely inhibit biofilm production, although most isolates (54.6%) significantly reduced biofilm production from strong or moderate (categories 2 and 3) to weak (category 1).

Suprainhibitory concentrations reduced the amount of preformed biofilm at all concentrations applied (*p* < 0.05). The effect was dose-dependent: a concentration of 2 × MIC completely eradicated biofilm in 12.1% of isolates and reduced the previously formed biofilm in 60.6% of isolates, and a concentration of 4 × MIC eradicated biofilm in 48.5% of isolates and reduced its category in 33.3% of isolates. The most effective concentration in eradicating the biofilm was again 8 × MIC, which resulted in complete eradication of the biofilm in 84.9% of the isolates and reduction of its category in all others (*p* < 0.05).

## 3. Discussion

NAC and propolis are two compounds that have been studied in fixed combination for their potential synergistic effects on oxidative stress and inflammatory or biochemical parameters in acute and chronic respiratory infections [21,22]. However, no data on synergism against bacterial biofilms have been published yet.

The efficacy of NAC on planktonic and sessile forms of *P. aeruginosa* strains isolated from patients with chronic suppurative otitis media was demonstrated in vitro at concentrations of 0.25% (2.5 mg/mL), 0.5% (5 mg/mL), and 1.25% (12.5 mg/mL) [23]. Similar results were obtained with *S. pneumoniae* and *H. influenzae* strains isolated from a child with acute otitis media, with complete elimination of planktonic bacteria at an MIC of 2.5 mg/mL of NAC [24], and with mixed biofilms of *S. pneumoniae* and MSSA or MRSA at an MIC between 2.5 mg/mL and 5 mg/mL [25]. A higher MIC (20 mg/mL) was found in MRSA and quinolone-resistant *P. aeruginosa* isolated from patients with post-tympanostomy tube otorrhea [26], and in *P. aeruginosa* strains isolated from other respiratory specimens (10–40 mg/mL) [27]. Our results are consistent with those of other studies, as the MIC for NAC alone ranged from 2.5–80 mg/mL, and in combination with propolis was 1.25–20 mg/mL. Although Gram-positive bacteria had lower MIC (<5 mg/mL) compared to Gram-negative bacteria (≥7.5 mg/mL), no statistically significant difference was found between these two groups of bacteria. Chlumsky et al. also concluded that the antimicrobial and antibiofilm effects of NAC are not related to cell wall structure, as NAC inhibits both Gram-positive and Gram-negative bacteria [28].

The antibacterial activity of propolis has already been studied and indicates that propolis has superior activity against Gram-positive compared to Gram-negative bacteria [29,30,31,32]. In our study, the MIC for dry propolis extract was in the range of 0.25–8 mg/mL, and the highest antimicrobial effect was observed in Gram-positive bacteria. Moreover, our results showed that the dry propolis extract was also effective against Gram-negative bacteria, with slightly higher MIC compared to Gram-positive bacteria. The MIC values are close to those reported by other authors [29,30,31,32], although the absolute values cannot be directly correlated, since we used a dry propolis extract standardized to 12% of total polyphenols, whereas others have mainly used a liquid ethanolic propolis extract of different geographical origin prepared according to the official standards for extract production.

The results of our study indicate that NAC/dry propolis extract had a synergistic effect in more than 50% of strains, as the MIC of each component in its fixed combination was reduced two- to fourfold compared with the MIC of each individual compound. Another positive pharmacological interaction, an additive effect was observed in almost 40% of the strains, and only 10% were indifferent to this combination. The inhibitory effect of NAC against Gram-positive and Gram-negative bacteria isolated from upper respiratory tract infections was reported in the range of 2.5 to >40 mg/mL [23,24,25,26,27]. According to our results, the MIC for NAC was reduced as a result of synergistic interactions and is lower compared to the above results. On the other hand, the MIC values for propolis vary depending on the solvent. Ethanolic and methanolic extracts have superior antimicrobial activity for both Gram-positive and Gram-negative bacteria compared to non-alcoholic extracts (water, DMSO, hexane). The antimicrobial activity of various propolis extracts was extensively reviewed by Przybyłek and Karpinski and the MIC values were reported as follows: *S. aureus* up to 3.1 mg/mL, *S. epidermidis* up to 1.1 mg/mL, *S. pneumoniae* up to 1.5 mg/mL, *P. aeruginosa* 7.9 mg/mL, and *H. influenzae* 2.5 mg/mL [14]. In our study, the MIC for propolis in fixed combination was reduced to 0.125–2 mg/mL, which can be attributed to similar synergistic effects as for NAC and confirms the beneficial effect of this specific NAC/propolis combination. This finding is of particular importance because the fixed combination of NAC and propolis can be used not only via oral route, but also as a local mucosal therapy, and lower concentrations of both compounds are less irritating to mucous membranes and well tolerated.

The specific mechanisms of synergistic effect between NAC and propolis may vary depending on the species of microorganisms or health conditions they target. Combining propolis with NAC may enhance their individual antimicrobial properties, potentially leading to a more effective antimicrobial clearance. Both compounds have immunomodulatory properties [11] and may have synergistic effects in promoting a balanced immune response and enhancing immune defense mechanisms, as well as antioxidative properties [33,34] that provide synergistic antioxidant effects, with enhanced protection against oxidative damage. All mentioned mechanisms have beneficial effects in vivo, as they reduce mucosal inflammation and damage and accelerate tissue regeneration.

In our study, the strong-biofilm producers were isolated from tissue samples of approximately 20–30% of patients; most of them belonged to the species *S. aureus*, *S. epidermidis*, and *P. aeruginosa*, depending on the specific pathology. The effect on NAC/dry propolis fixed combination on biofilm formation and eradication of biofilms was dose-dependent and more pronounced in strong biofilm producers.

The first report on the effect of NAC on biofilm formation was published in 1997 by Perez-Giraldo et al. [35]. Although a different method than ours was used for biofilm determination, inhibition of biofilm formation by more than 50% was observed in 15 strains of *S. epidermidis* when NAC was applied at concentrations of 1–8 mg/mL, and concentrations above 0.25 mg/mL significantly reduced biofilm OD [35]. A recent study described in detail the mechanisms of antibiofilm effect of NAC as a single treatment or in combination with other enzymes and antibiotics on MRSA and MSSA biofilms [36]. The authors confirmed that NAC exhibited a complete bacteriostatic effect on planktonic *S. aureus* bacteria and inhibited EPS polysaccharide production in all strains of staphylococci tested, with a particularly pronounced effect on MRSA biofilms at a concentration of 30 mM NAC (approximately 4.9 mg/mL). In addition, a concentration of 1 mM resulted in complete cleavage of dsDNA in the biofilms reducing eDNA, which is an essential component of staphylococcal biofilms. One possible mechanism for the effect of propolis on staphylococcal biofilms is the inhibition of quorum sensing in staphylococci [37]. The effect of 30% ethanolic propolis extract on inhibition and disruption of biofilms in staphylococci was recently reported by Queiroga et al. [38]. The inhibitory effect on biofilm formation of *S. aureus* and various CNS species (*S. epidermidis*, *S. chromogenes*, *S. warneri*, *S. auricularis*, *S. simulans*, *S. caprae*, *S. capitis*) was observed at subinhibitory concentrations of 1/2 MIC, and suprainhibitory concentrations of 1/2 MBC resulted in biofilm disruption. However, the authors tested only one concentration for biofilm inhibition and one for eradication. Other authors also reported antibiofilm activity of propolis against *S. epidermidis* [39], *S. aureus* [40], and MSSA [41]. Although our results of the biofilm-inhibitory effect of NAC and propolis on staphylococci are in agreement with those of other authors, we must point out that only *S. aureus* isolates and one CNS strain (*S. epidermidis*) were strong biofilm producers and inhibition of biofilm formation/biofilm disruption was observed at all concentrations tested.

In 20 clinical isolates of *P. aeruginosa* isolated from respiratory samples, a dose-dependent dissolution of mature biofilms and a reduction in EPS production were also observed at NAC concentrations of 0.5–2.5 mg/mL, and biofilm detachment was observed at higher concentrations (10 mg/mL) [27]. In addition to NAC, a reduction in *P. aeruginosa* biofilm biomass was observed when 50 and 100 μg/mL of ethanolic propolis extract were applied, although the effect was not due to a reduction in EPS but to a reduced number of sessile bacteria, as confirmed by the reduction in *P. aeruginosa* luminescence [42]. The *P. aeruginosa* isolates tested in our study were capable of producing greater amounts of biofilm, and the NAC/propolis combination had biofilm inhibitory and biofilm eradicative effects at concentrations similar to those mentioned above. This could be the result of the synergistic effect of the NAC/propolis combination on dissolving EPS and reducing the number of viable cells in the biofilm, as the sulfhydryl group of NAC reacts with disulfide bonds in enzymes involved in EPS production or excretion, thereby reducing EPS, which in turn allows propolis penetration into the biofilm and its antibacterial effect on sessile cells.

Of the other bacterial species examined in our study, isolates of *H. influenzae*, *M. catarrhalis*, and *S. pneumoniae*, obtained from patients with chronic otitis media and chronic adenoiditis, formed lower amounts of biofilm. Although lower amounts of biofilm are considered easier to treat, these bacterial species tend to form polymicrobial biofilms with other pathogens, or members of the normal mucosal microbiota. An investigation of the in vitro effect of NAC on the biofilm production of two strains with large biofilm production capacity, *S. pneumoniae* and *H. influenzae* isolated from a child with acute otitis media, and their mixed biofilms, confirmed that NAC at a concentration of 0.5 mg/mL killed 99% of *S. pneumoniae* cells and eliminated all *H. influenzae* cells, and that a concentration of 2.5 mg/mL (i.e., the MIC for planktonic cells) eradicated biofilms [24]. The antimicrobial activity of various propolis extracts against *S. pneumoniae* and *H. influenzae* was reported at concentrations of 0.08–0.6 mg/mL and 0.6–2.5 mg/mL, respectively, but no data on their antibiofilm activity were evaluated [43]. Similarly, Drago et al. reported that *S. pneumoniae*, *H. influenzae*, and *M. catarrhalis* were more sensitive to dry propolis extract than *S. aureus*, with MIC values in the range of 0.031–1 mg/mL [29]. The antimicrobial activity of the dry propolis extract tested in our study was in the range of 0.375–1.5 mg/mL when combined with NAC, which is very similar to the results reported by Al-Ani et al. [43] and Drago et al. [29]. In addition to the bactericidal effect on biofilm-forming cells, reduction of EPS, and disruption of biofilm, other authors suggested that the beneficial effect of NAC on biofilm formation by respiratory pathogens may be the result of direct inhibition of bacterial attachment to respiratory epithelial cells. This has been reported in several in vitro studies on human oropharyngeal epithelial cells isolated from healthy volunteers and patients with chronic pulmonary disease (chronic bronchitis, chronic emphysema, chronic bronchial asthma, and bronchiectasis) [44,45].

NAC is usually administered orally (200 to 600 mg two or three times daily), and the concentrations achieved in oropharyngeal secretions are very close to those that have been described as biofilm inhibitory [44]. In addition to oral administration, NAC can also be administered intravenously, achieving higher peak plasma concentrations, and by nebulization or direct instillation with higher local concentrations that inhibit biofilm formation or disrupt already formed biofilms [35]. Another way to increase local concentrations in the middle ear is by trans-tympanic NAC injections, which have been shown to be safe in humans [46]. Conversely, therapeutic recommendations of the use of propolis are not clearly defined, and no recommendations or monographs can be found in cPh Eur. A recent study reviewed the use of propolis in dentistry and oral medicine, but the exact dosage and route of administration of propolis are still not defined in official guidelines [47].

It should be noted that the antibiofilm activity of propolis may vary depending on the specific composition of the propolis, its geographical origin, and the microbial species involved. In addition, further research is needed to fully understand the mechanisms and optimize the use of NAC in combination with propolis as an antibacterial and antibiofilm agent in upper respiratory tract infections.

## 4. Materials and Methods

### 4.1. Study Design and Population

This prospective study was conducted from February until June 2023. at the Clinic of Otorhinolaryngology and Maxillofacial Surgery, University Clinical Centre of Serbia as a tertial referral center. The study was approved by the Ethics Committee of the University of Belgrade—Faculty of Medicine, Belgrade, Serbia (No.29/XII-30, 1 December 2014) and the Ethics Committee of the University Clinical Centre of Serbia, Belgrade, Serbia (17-6/2023, 26 January 2023). All patients or their parents/legal guardians signed an informed consent form before inclusion in the study.

The study included 48 adults with chronic rhinosinusitis, 29 adults with chronic otitis media, and 33 children with chronic adenoiditis, who were treated surgically according to the indication for each condition. Patients underwent a preoperative evaluation that included a detailed history and otorhinolaryngologic examination with otomicroscopy and nasal endoscopy. Demographic data of the patients included in the study were recorded. Preoperative additional imaging was performed, namely computed tomography (CT) of the temporal bones in patients with chronic otitis media or of the paranasal sinuses in patients with chronic rhinosinusitis. Exclusion criteria were the presence of other chronic respiratory diseases (asthma, allergic rhinitis, cystic fibrosis) and congenital malformations of the head and neck, previous surgical procedures (middle ear surgery, paranasal sinus surgery or adenoidectomy) and upper respiratory tract infections treated with antibiotics 7–14 days before the procedure.

### 4.2. Specimens’ Collection and Bacterial Isolation and Identification

In patients with chronic rhinosinusitis, a sample of sinus mucosa was obtained from the upper parts and roof of the ethmoid sinus during surgery. In all children with chronic adenoiditis included in the study, adenoid tissue was collected during adenoidectomy. In patients with chronic otitis media, middle ear mucosa was harvested from the mastoid cavity and tympanic cavity during surgery.

Tissue samples collected from each patient were processed and analyzed in the microbiology laboratory within 2 h after collection. Each sample was washed thoroughly in three separate beakers of sterile saline to remove all planktonic bacteria. Samples were homogenized in sterile saline using a sterile glass/Teflon Potter Elvehjem homogenizer, (Thomas Scientific LLC, Swedesboro, NJ, USA). The homogenate was inoculated into brain-heart infusion broth (Thermo Scientific™ Remel BHI Broth, ThermoFisher Scientific, Waltham, MA, USA, Catalog No. R060270) and incubated at 37 °C for 24 h. Chocolate agar (bioMérieux, Marcy-l’Etoile, France, Ref. 43101) or blood agar (bioMérieux, Marcy-l’Etoile, France, Ref. 43321) and MacConkey agar (bioMérieux, Marcy-l’Etoile, France, Ref. 43141) were streaked with the 24 h cultures and incubated under the same conditions. Strain identification was performed using MALDI-TOF Vitek MS System (bioMérieux, Marcy-l’Etoile, France).

### 4.3. Determination of Minimum Inhibitory Concentration of NAC/Dry Propolis Extract Fixed Combination

The antimicrobial activity of the commercially available fixed combination of NAC and dry propolis extract standardized to 12% of total polyphenols (200 mg NAC/80 mg dry propolis extract, AbelaPharm, Belgrade, Serbia) was determined by broth microdilution assay in 96-well microtiter plates according to the European Committee for Antimicrobial Susceptibility Testing guidelines http://www.eucast.org (accessed on 3 July 2023).

Bacterial suspensions were prepared in sterile saline (bioMérieux, Marcy-l’Etoile, France, Ref. 70770) to a density of 0.5 McFarland standard (McFarland Densimat densitometer, bioMérieux, Marcy-l’Etoile, France), and further diluted to an inoculum containing 5 × 10^5^ CFU/mL of microorganisms. All tested compounds were dissolved in fresh Mueller-Hinton broth (Thermo Scientific™ MHB, ThermoFisher Scientific, Waltham, MA, USA, Cat. No. CM0405B) and prepared in double dilutions (1:4–1:64) to working concentrations in the range of 0.62/0.06–80/8 mg NAC/mg propolis per milliliter. Each concentration was used in triplicate in 96-well flat-bottom polystyrene microtiter plates (Nunc™ MicroWell™ 96-Well Microplates, ThermoFisher Scientific, Waltham, MA, USA, Cat. No. 243656) and inoculated with 5 × 10^5^ CFU/mL of microorganisms. To detect bacterial viability (i.e., activity of cellular dehydrogenase in metabolically active cells), 0.05% 2,3,5-triphenyl-2H-tetrazolium chloride (Sigma-Aldrich-Merck KGaA, Darmstadt, Germany, Cat. No. 17779) was added to MHB as a redox indicator. Minimum inhibitory concentrations (MICs) were determined as the lowest concentration of the tested compounds that inhibited bacterial growth after incubation for 18 ± 2 h at 35 ± 1 °C under aerobic or capneic conditions. Positive growth controls (microorganisms in the medium) and negative controls (medium with compounds) were established for each strain and compound tested, and each test was repeated three times.

### 4.4. Synergy Testing

To determine whether each component of NAC/dry propolis extract fixed combination alone, or in combination, exhibited better antimicrobial activity, their pharmacological interaction was investigated in 96-well flat-bottom polystyrene microtiter plates (ThermoFisher Scientific, Waltham, MA, USA, Cat. No. 243656) using the checkerboard method [48]. First, the MICs for NAC (AbelaPharm, Belgrade, Serbia) and dry propolis extract standardized to 12% of total polyphenols (AbelaPharm, Belgrade, Serbia) were determined as previously described. After determining the MIC, the synergistic effect of their combination was investigated in one concentration above the MIC (2 × MIC) and five concentrations below the MIC (1/2–1/32) of each compound. The type of pharmacological interaction between two tested compounds was estimated by calculating fractional inhibitory concentration (FIC) and fractional inhibitory concentration indices (FICI). The FIC of each compound was calculated by dividing the concentration of the compound in the effective MIC of the combination, by the MIC of the drug alone (e.g., FIC_NAC_ = MIC_NAC-propolis combination_/MIC_NAC_). FICI values were calculated as the sum of the FIC_NAC_ and FIC_propolis_ and interpreted as follows: FICI ≤ 0.5 synergy; 0.5 < FICI ≤ 4 indifference (no interaction) and FICI > 4.0 antagonism [49]. Each test was repeated three times.

### 4.5. In Vitro Determination of Bacterial Biofilm-Forming Capacity

The biofilm producing capacity of the bacterial isolates was tested in 96-well flat-bottom polystyrene microtiter plates (ThermoFisher Scientific, Waltham, MA, USA, Cat. No. 243656) according to Stepanović et al. [50]. Bacteria were incubated for 24 h as previously described. A 180 μL of tryptic soy broth (Thermo Scientific™ Remel TSB, ThermoFisher Scientific, Waltham, MA, USA, Cat. No. R117835) was added to the microtiter plate in triplicate, and 20 μL of freshly prepared bacterial suspension (1.5 × 10^8^ CFU/mL) was added to each well. The positive control of each strain consisted of a triplicate of bacteria cultured only in the medium, and two triplicates of the medium alone were the negative control of each plate.

Plates were incubated for 24 h at 35 ± 1 °C under aerobic or capneic conditions, then washed with PBS (ThermoFisher Scientific, Waltham, MA, USA, Cat. No. 10010023) and air-dried. The biofilm was fixed with methanol and stained with 2% crystal violet (Sigma-Aldrich-Merck, Cat. No. C0775). The dye was released with 96% ethanol (Sigma-Aldrich-Merck, Cat. No. 1590102500) and the presence of biofilm was measured at an optical density of 570 nm (Multiskan™ FC Microplate Photometer, ThermoFisher Scientific, Waltham, MA, USA). The category of biofilm production was calculated in relation to the cut-off value (ODc; as the mean OD value of the negative control +3 SD) and expressed as follows: category 0: OD ≤ ODc—no biofilm production; category 1: ODc < OD ≤ 2 × ODc—weak biofilm production; category 2: 2 × ODc < OD ≤ 4 × ODc—moderate biofilm production; category 3: 4 × ODc < OD—strong biofilm production.

### 4.6. Effect of NAC/Dry Propolis Extract Combinations on Biofilm Formation and Biofilm Eradication

The effect of NAC/dry propolis extract fixed combination on biofilm formation was investigated in five concentrations below the MIC, and the biofilm-eradication in three concentrations above the MIC.

To test the effect of NAC/dry propolis extract fixed combination on biofilm formation, serial dilutions of the compounds ranging from 1/2 to 1/8 MIC were prepared in TSB (ThermoFisher Scientific, Waltham, MA, USA, Cat. No. R117835). A 180 μL of each dilution and 20 μL of the bacterial suspension were added in triplicate to the 96-well flat-bottom polystyrene microtiter plates (ThermoFisher Scientific, Waltham, MA, USA, Cat. No. 243656). The positive and negative controls were also added to each plate. The bacteria were incubated for 24 h at 35 ± 1 °C under aerobic or capneic conditions, and the amount of biofilm was measured as described previously.

To test the effect of NAC/dry propolis extract fixed combination on the eradication of the previously formed biofilm, bacteria were preincubated in TSB (Thermo Scientific™ Remel TSB, ThermoFisher Scientific, Waltham, MA, USA, Cat. No. R117835) for 24 h at 35 ± 1 °C under aerobic or capneic conditions, and plates were rinsed with sterile phosphate-buffered saline (ThermoFisher Scientific, Waltham, MA, USA, Cat. No. 10010023). Serial dilutions of the compounds ranging from 2 × MIC to 8 × MIC were prepared in TSB (Thermo Scientific™ Remel TSB, ThermoFisher Scientific, Waltham, MA, USA, Cat. No. R117835), and 200 μL of each dilution was added in triplicate to the 96-well flat-bottom polystyrene microtiter plates (ThermoFisher Scientific, Waltham, MA, USA, Cat. No. 243656) containing the previously formed biofilm. Positive and negative controls were used for each plate as previously described. The plates were incubated for an additional 24 h, and the biofilms were detected as described above.

### 4.7. Statistical Analysis

The statistical program SPSS (PASW Statistics for Windows, Version 18.0: SPSS Inc., Chicago, IL, USA) was used for statistical analysis. Considering the expected incidence in a study group, the required sample size of 28 patients with chronic suppurative otitis media, 44 patients with chronic sinusitis, and 30 patients with chronic adenoiditis was determined based on data from previously published studies with the frequency of occurrence of these diseases in the known population (3.4%, 12%, and 34%, respectively) at a significance level α = 0.05 (5%) and the statistical power of the test 1 − β = 0.8 (80%). The total number of patients recruited for the study was then estimated to be 102. The data obtained in this study were analyzed by the methods of descriptive statistics, Chi square test, Student’s *t*-test (post-hoc pairwise, with Bonferroni adjustment) and Mann-Whitney U test.

## 5. Conclusions

In conclusion, subinhibitory concentrations of NAC/dry propolis fixed combination inhibited biofilm formation in all tested strains of bacteria. Although our results agree with those of other authors, we must emphasize that the mean biofilm inhibitory concentrations of NAC in combination with propolis were much lower than the concentrations used by other authors when NAC was applied alone. The same results were observed for the biofilm eradication activity of NAC/dry propolis fixed combination, as suprainhibitory concentrations completely eradicated biofilm in the majority of isolates. This is the major advantage of NAC/dry propolis fixed combination, as these concentrations can be easily achieved by local administration at the site of infection. Further studies are needed to investigate the optimal dosages, formulations, and clinical efficacy of using NAC and propolis together in the treatment of biofilm-related chronic upper respiratory tract infections.

## Data Availability

Data is contained within the article and Supplementary Material.

## Supplementary Materials

The following supporting information can be downloaded at: https://www.mdpi.com/article/10.3390/ph16111604/s1, Table S1: MALDI-TOF MS identification of isolated bacteria.
