# Supplementary material for: In Vitro Antibiofilm Effect of N-Acetyl-L-cysteine/Dry Propolis Extract Combination on Bacterial Pathogens Isolated from Upper Respiratory Tract Infections
Source: Pharmaceuticals (Basel). 2023 Nov 14;16(11):1604. doi: 10.3390/ph16111604 (PMC10674846; doi:10.3390/ph16111604)
Supplement: Supplementary file 1 [file pharmaceuticals-16-01604-s001.zip › pharmaceuticals-2657683-supplementary.pdf]

Supplementary materials

Table S 1. MALDI-TOF MS identification of isolated bacteria.

Isolate code number/MALDI TOF identification		
**Chronic rhinosinusitis**	**Chronic otitis media**	**Chronic adenoiditis**
1. *S. aureus*	49. *H. influenzae*	84. *H. influenzae*
2. *S. aureus*	50. *H. influenzae*	85. *H. influenzae*
3. *S. aureus*	51. *H. influenzae*	86. *M. catarrhalis*
4. *S. aureus*	52. *H. influenzae*	87. *M. catarrhalis*
5. *S. aureus*	53. *M. catarrhalis*	88. *M. catarrhalis*
6. *S. aureus*	54. *M. catarrhalis*	89. *M. catarrhalis*
7. *S. aureus*	55. *M. catarrhalis*	90. *M. catarrhalis*
8. *S. aureus*	56. *M. catarrhalis*	91. *M. catarrhalis*
9. *S. aureus*	57. *M. catarrhalis*	92. *M. catarrhalis*
10. *S. aureus*	58. *P. aeruginosa*	93. *P. aeruginosa*
11. *S. aureus*	59. *P. aeruginosa*	94. *P. aeruginosa*
12. *S. aureus*	60. *P. aeruginosa*	95. *S. aureus*
13. *S. aureus*	61. *P. aeruginosa*	96. *S. aureus*
14. *S. epidermidis*	62. *P. aeruginosa*	97. *S. aureus*
15. *S. epidermidis*	63. *P. aeruginosa*	98. *S. aureus*
16. *S. epidermidis*	64. *P. aeruginosa*	99. *S. aureus*
17. *S. epidermidis*	65. *S. aureus*	100. *S. aureus*
18. *S. epidermidis*	66. *S. aureus*	101. *S. aureus*
19. *S. epidermidis*	67. *S. aureus*	102. *S. aureus*
20. *S. epidermidis*	68. *S. aureus*	103. *S. aureus*
21. *S. epidermidis*	69. *S. aureus*	104. *S. epidermidis*
22. *S. epidermidis*	70. *S. aureus*	105. *S. epidermidis*
23. *S. epidermidis*	71. *S. aureus*	106. *S. epidermidis*
24. *S. epidermidis*	72. *S. aureus*	107. *S. epidermidis*
25. *S. epidermidis*	73. *S. epidermidis*	108. *S. epidermidis*
26. *S. epidermidis*	74. *S. epidermidis*	109. *S. haemolyticus*
27. *S. epidermidis*	75. *S. epidermidis*	110. *S. haemolyticus*
28. *S. epidermidis*	76. *S. epidermidis*	111. *S. pneumoniae*
29. *S. epidermidis*	77. *S. epidermidis*	112. *S. pneumoniae*
30. *S. haemolyticus*	78. *S. pneumoniae*	113. *S. pneumoniae*
31. *S. haemolyticus*	79. *S. pneumoniae*	114. *S. pneumoniae*
32. *S. haemolyticus*	80. *S. pneumoniae*	115. *S. pneumoniae*
33. *S. haemolyticus*	81. *S. pneumoniae*	116. *S. pneumoniae*
34. *S. haemolyticus*	82. *S. pneumoniae*	
35. *S. haemolyticus*	83. *S. pneumoniae*	
36. *S. lugdunensis*		
37. *S. warneri*		
38. *S. warneri*		
39. *Str. pneumoniae*		
40. *Str. pneumoniae*		
41. *Str. pneumoniae*		
42. *P. aeruginosa*		
43. *P. aeruginosa*		
44. *P. aeruginosa*		

45. *P. aeruginosa*		
46. *M. catarrhalis*		
47. *M. catarrhalis*		
48. *M. catarrhalis*		
